# A Scoping Review of Patient-Centered Perinatal Contraceptive Counseling

**DOI:** 10.1007/s10995-024-03946-y

**Published:** 2024-08-01

**Authors:** Jennifer Karlin, Rebecca L. Newmark, Nina Oberman, Christine Dehlendorf

**Affiliations:** 1 Family and Community Medicine, University of California, San Francisco, CA 94110 USA; 2 San Francisco School of Medicine, University of California, San Francisco, CA USA; 3 San Francisco Department of Humanities and Social Sciences, University of California, San Francisco, CA USA; 4 Berkeley School of Public Health, University of California, Berkeley, CA USA

**Keywords:** Contraceptive counseling, Patient preference, Patience experience, Patient-centered care, Shared decision-making

## Abstract

**Introduction:**

Contraceptive counseling during the perinatal period is an important component of comprehensive perinatal care. We synthesized research about contraceptive counseling during the perinatal period, which has not previously been systematically compiled.

**Methods:**

We developed search criteria to identify articles listed in PubMed, Embase, and Popline databases published between 1992 and July 2022 that address patients’ preferences for, and experiences of, perinatal contraceptive counseling, as well as health outcomes associated with this counseling. Search results were independently reviewed by multiple reviewers to assess relevance for the present review. Methods were conducted in accordance with PRISMA guidelines.

**Results:**

Thirty-four articles were included in the final full text review. Of the included articles, 10 included implementation and evaluation of a contraceptive counseling method or protocol, and 24 evaluated preferences for or experiences of existing contraceptive counseling in the perinatal period. Common themes included the acceptability of contraceptive counseling in the peripartum and postpartum periods, and a preference for contraceptive counseling at some point during the antenatal period and before the inpatient hospital experience, and direct provider-patient discussion instead of video or written material. Multiple studies suggest that timing, content, and modality should be individualized. In general, avoiding actual or perceived directiveness and providing multi-modal counseling that includes both written educational materials and patient-provider conversations was desired.

**Discussion:**

The perinatal period constitutes a critical opportunity to provide contraceptive counseling that can support pregnant and postpartum people’s management of their reproductive futures. The reviewed studies highlight the importance of patient-centered approach to providing this care, including flexibility of timing, content, and modality to accommodate individual preferences.

## Significance

Our scoping review confirms that patients are receptive to contraceptive counseling in the perinatal period, and that this counseling should be delivered in an individualized manner given the diversity of patient needs during pregnancy and postpartum. More research is required to implement protocols that are flexible and patient centered.

## Background

Meeting individuals’ health needs in the perinatal period includes provision of quality contraceptive counseling and care. In addition to supporting people to create the families they desire, the provision of quality contraceptive care also can help prevent short interpregnancy intervals (six months or less), which are associated with preterm birth, low birth weight, and gestational diabetes (Conde-Adudelo A. et al., 2006; Hanley GE et al., 2017). The perinatal period is considered the time between when the patient knows that they are pregnant until a year following delivery. Despite the importance of meeting people’s needs for contraceptive care in the perinatal period, nearly half of perinatal patients report never discussing postpartum contraception with their health care provider during prenatal care (Weisband et al., 2017). Over the past decade, there has been a significant shift in the in understanding of best practices for contraceptive counseling in reproductive health care generally, with a transition from a clinician-centric model that prioritizes efficacy and clinician-controlled methods to one which focuses more on patient-centered care and approaches counseling from a framework of reproductive justice. Reproductive justice is defined as “the human right to maintain personal bodily autonomy, have children, not have children and parent the children we have in safe and sustainable communities” (Ross, 2017). As the American College of Obstetricians and Gynecologists (ACOG) outlines in a 2022 committee statement, “Ob-gyns should incorporate the reproductive justice framework into contraceptive counseling by acknowledging the historical and ongoing mistreatment of… marginalized individuals whose reproductive desires have been devalued; recognize counselor bias…and prioritizing patients’ values, preferences and lived experience in the selection or discontinuation of a contraceptive method” (ACOG, 2022).

While the shift in understanding of and provision of counseling has been notable in contraceptive care generally, less attention has been paid to contraceptive care specific to the perinatal period. As this period is unique with respect to social and physiological transitions, understanding the experiences and preferences for perinatal contraceptive counseling, as well as how this counseling is associated with health outcomes, is critical. This is particularly true given that non-patient-centered and paternalistic care are well documented to occur during perinatal care, particularly for those with minoritized racial/ethnic identities. (Akinade et al., 2023; Altman et al., 2020; Bohren et al., 2022; Hamed et al., 2022; Hemphill et al., 2023; Liese et al., 2021; Logan et al., 2022; Thompson et al., 2022). Exploring the literature about perinatal contraceptive counseling applying a person-centered and reproductive justice-aligned lens can inform future work to ensure that pregnant people and those who have recently given birth are supported in their reproductive decision making.

In this scoping review, we aim to understand patients’ experiences with, and preferences for, as well as health outcomes associated with, contraceptive counseling during the perinatal period. We synthesize existing research on counseling interventions delivered during this period to propose best practices, discuss gaps in research, and assess if the literature reflect the movement towards patient-centeredness and reproductive justice in contraceptive counseling literature and practice.

## Methods

### Search Strategy

Our scoping review methodology followed frameworks developed by Arskey & O’Malley (2005) and Levac et al. (2010), as well as Preferred Reporting Items for Systematic Reviews and Meta-Analyses (PRISMA-ScR) guidelines (Rethlefsen et al., 2019; Tricco et al., 2018) (Appendices 1 & 2). We registered our review in PROSPERO under #CRD42020134001. This research was conducted in accordance with prevailing ethical principles and was not reviewed by an Institutional Review Board given that it was an analysis of previously published material.

**Fig. 1:**
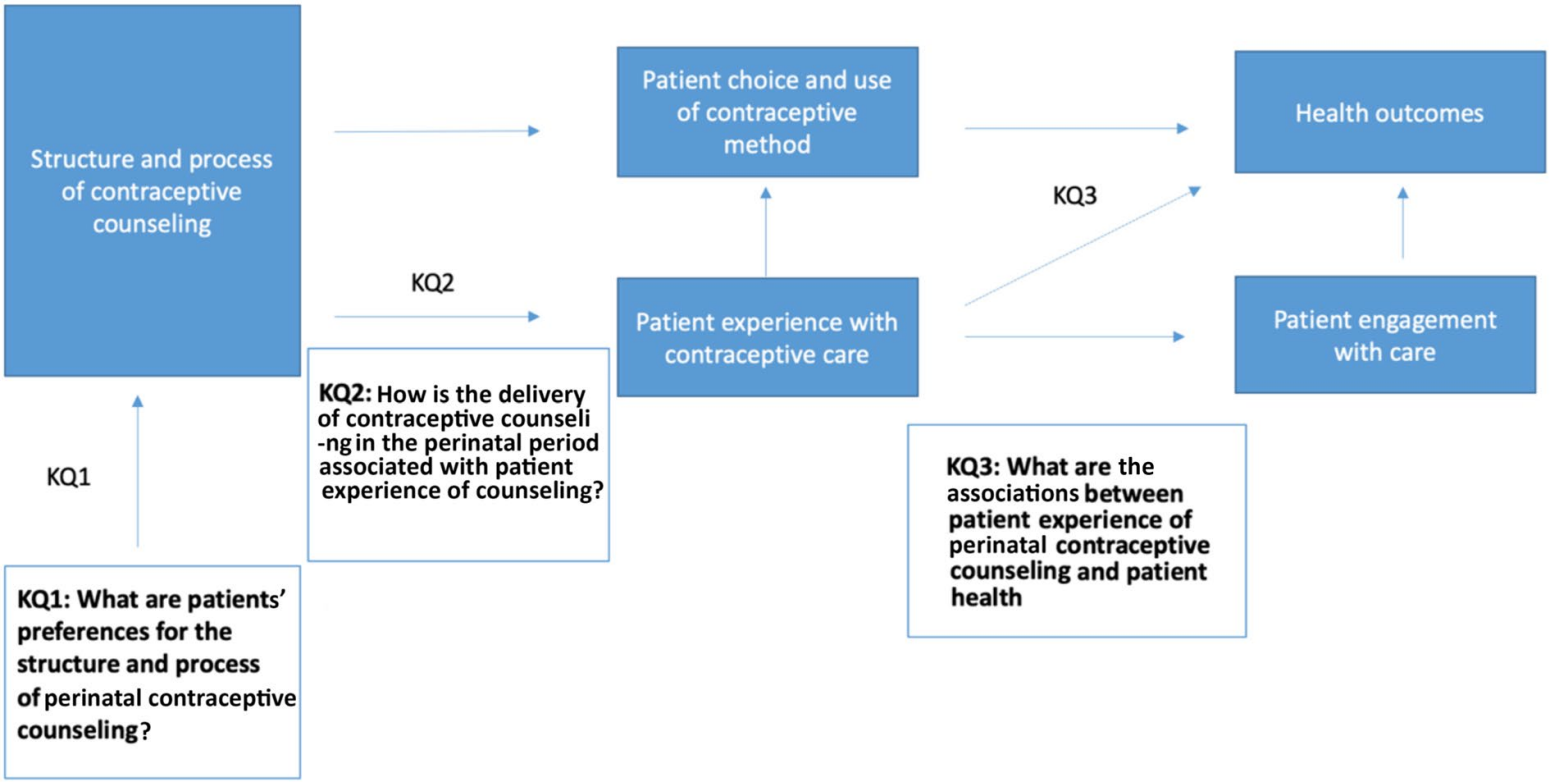
Analytic framework for systematic review of contraceptive counseling and education (KQ: Key Question)

We modified the Office of Population Affairs (OPA) conceptual framework for clinical performance measures for contraceptive care (Gavin et al., 2017) to create our analytic framework, as shown in Fig. 1. We focused on three key questions (KQ) that lie at the connections between the structure and process of care and outcomes that result from use of contraceptives during the postpartum period. Our key questions include the following: KQ1: What are patients’ preferences for the structure and process (i.e. who should be doing the counseling, when it should occur and what it should include) of perinatal contraceptive counseling? KQ2: How is the delivery of contraceptive counseling in the perinatal period associated with patient experience of counseling? And KQ3: What are the associations between patients’ experiences of perinatal contraceptive counseling and health outcomes? We did not consider contraceptive use as a health outcome; rather, studies had to measure clinically relevant health outcomes that affected the pregnant person or fetus. The articles included in this scoping review all addressed at least one of these key questions.

We used a three-step search process to identify studies for this review. First, we harvested terms by identifying keywords and controlled vocabulary, including MeSH and Emtree terms, from key articles on our topic. Next, we developed a search strategy in collaboration with a clinical librarian using an iterative process that involved testing search terms and examining the relevance of corresponding search results. Our search combined the concepts of contraception and the perinatal period with our three key questions. Boolean logic was applied by combining similar keywords and controlled vocabulary with OR and using AND between each concept: for example, (“Contraception Behavior”[Mesh] OR contraceptives) AND (Peripartum Period”[Mesh] OR postpartum) AND (perceptions OR satisfaction OR “low birth weight”).

We conducted a systematic search in two waves. Our first search was conducted with PubMed, Embase, and Popline on June 17, 2019 and was limited to 1992–2019. Our second search was conducted with Embase and PubMed to include articles published from June 17, 2019-July 1, 2022 to update the articles (Popline had been discontinued in the interim). We chose 1992 as a starting point in the literature to reflect the advances in contraception and patient-centered care that occurred after this date, specifically the FDA approval of depot medroxyprogesterone acetate (DMPA) in 1992. We defined the perinatal period as the time between pregnancy and one year following delivery. Within this perinatal period, authors also use the term peripartum period to define the time immediately before, during, and after delivery; the antenatal period is defined as immediately prior to the delivery and not including the delivery; and the postpartum period is defined as immediately and up to one year following delivery. No language limits were used. Detailed search strategies for each database can be found in Appendix 3. Finally, cited reference searching was conducted by two reviewers using the reference lists of all included articles to identify additional relevant studies.

### Study Selection

Using Covidence, an online data management system for systematic reviews, two reviewers (JK, RLN) independently screened all articles based on title and abstract, and three reviewers independently screened for full-text review (JK, RLN, NO). Reviewers collaboratively reviewed screening decisions at each stage to ensure agreement. In accordance with previous systematic reviews on contraceptive counseling (Fox et al., 2018), studies were excluded if they: (1) did not contain the full text of the article; (2) were not in English; (3) were not based in a setting that included the following locations: US, UK, Australia, Europe, New Zealand, or Canada (we based this decision in alignment with previous studies illustrating that contraceptive counseling and access to birth control methods are substantially different in low and middle income countries, the most recent of which includes Ross et al., 2023); (4) did not assess patient preferences for or experiences of contraceptive counseling; or (5) did not include a population of reproductive age patients receiving services in a clinical setting during the perinatal period. We included grey literature given that we wanted to do a scoping review that was inclusive of all materials that discussed preferences, given that the peer-reviewed literature has had limited engagement with patient preferences in this context. Systematic reviews were excluded but their citations were reviewed to ensure we had not missed any publications in our initial query.

### Data Extraction

We created a standardized form to extract data in the following broad areas: 1) study design & setting; 2) study population & demographics; 3) patient preferences and experiences around contraceptive counseling; 4) timing, location and type of provider that enacted intervention; 5) short-term and/or adverse clinical outcomes, and 6) health outcomes. In accordance with scoping review methodology, critical appraisal was not conducted (Arskey & O’Malley, 2005; Levac et al., 2010). Data extraction was completed by two reviewers in each wave (RLN, NO in the first wave and RLN and JK in the second) with all articles reviewed by a third (JK).

To be included in the review, articles had to answer at least one of our three key questions. Articles were mapped onto key questions as summarized in Tables 1 and 2 and the evidence synthesis subsections below.

**Table 1:** Design and key findings for studies that implemented a new contraceptive counseling protocol (N = 10)

Author (year)	KQ1	KQ2	KQ3	Timing of Contraceptive Counseling	Study Location	Special Population	N (patients)	Study Design and key findings
Frarey (2019)				Immediate postpartum prior to hospital discharge	Philadelphia, PA, USA	Adolescents	100	**RCT** -Standardized pp counseling (control) vs counseling incorporating information on health birth spacing and LARC methods + standardized postpartum counseling (intervention). -Assessed differences in repeat pregnancy, contraceptive initiation, continuation, and satisfaction (with method) and found no difference in satisfaction rates (also no difference in rate of initiating or type of contraception initiated).
Gallagher (2019)				Antenatal	Family Nurse Partnership (FNP) program clients in Scotland, UK	First time pregnancies for adolescents	118	**Cohort** -Evaluate intervention of antenatal contraceptive counseling and provision of postpartum contraception provided by midwives for those enrolled in FNP program and assessed timing and satisfaction of contraceptive counseling. - Found antenatal contraceptive counseling “helpful” compared to none, and timing “about right” at 22 weeks.
Haider (2020)				6 week WCC	Urban academic medical center, USA	No	446	**RCT** -Evaluate if offering co-located contraceptive services at well-baby visit increases use of LARC at 5 months pp and experience of counseling.
Johnson (2003)				Immediate postpartum period	Portland, Oregon, USA	No	109	**Cohort** -Baseline satisfaction and effectiveness of counseling (control) compared with pts who received additional comprehensive written educational material (intervention). -Low uptake, but of those who accepted contraceptive visit, 70% received new birth control method and 80% satisfied with contraceptive counseling although only 64% would recommend to friend. -Recommended scheduling with pediatric appointment.
Kumaraswami (2018)				Well Baby Visit: postpartum, 0–12 weeks	Urban Academic Medical Center, USA	No	200	**Cross-sectional** -Compared contraceptive counseling at postpartum visit (control) vs well baby visit (intervention). -Higher satisfaction of contraceptive counseling paired with well-baby visit compared to postpartum visit.
Moniz (2022)				Immediate postpartum	Academic medical center, USA	No	425	**Implementation and cross-sectional** -Evaluate feasibility of toolkit-based implementation of immediate postpartum LARC counseling and provision and patient experience found no improvement of satisfaction regarding contraceptive counseling.
Proctor (2006)				Immediate postpartum period (during hospitalization)	Charlotte, NC, USA	No	319	**RCT** -Pt Satisfaction with 3 pp counseling comparing physician–patient counseling vs written literature, vs educational video. -Assessed patient satisfaction with contraceptive counseling and found no difference among groups.
Reyes-Lacalle (2020)				30 week gestation prenatal visit	Public, primary care facilities with Midwives in Catalonia, Spain	No	975	**RCT** -Effectiveness of supplemental perinatal contraceptive counseling (intervention) in addition to standard Spanish postpartum counseling (control). -Assessed sexual activity, contraceptive use, and satisfaction of counseling and found that supplemental support resulted in higher effectiveness of contraceptive used in Spanish population but no relationship found between satisfaction and contraceptive method nor satisfaction with counseling or method choice.
Smith (2002)				Between 24 and 36 weeks gestation	Edinburgh, Scotland (and Shanghai and Cape Town)	No	329 in Scotland	**RCT** -Standard vs expert contraceptive advice from specialist nurse. -Assessed prevalence of contraceptive use and pregnancy rates at 1 year btw participants and controls and found that while all groups found contraceptive counseling “useful,” this did not change contraceptive use postpartum.
Staley (2019)				third trimester (28 + weeks)	Chapel Hill, NC, USA	No	84	**RCT** -Evaluated LARC-focused video (LARC FIRST) counseling during prenatal care vs standard care. -Assessed LARC uptake at 12 weeks and overall contraception use at 12 weeks postpartum, postpartum attendance, and acceptability of video counseling. -Participants reported satisfaction and increase of contraceptive knowledge from video, but this did not change LARC uptake.

*RCT* Randomized Control Trial; *LARC* Long-Acting Reversible Contraception

**Table 2:** Key findings and methods for studies evaluating patient experience of routine contraceptive counseling (N = 23)

Author (year)	KQ1	KQ2	KQ3	Study Location	Special Population	N (patients)	Study Design/ pt experience outcome assessed	Key findings about patient experience of contraceptive counseling
Cameron (2017)				22-week antenatal appointment midwifery clinic; Scotland UK	No	794 survey, 1369 medical records	**Cohort, database review and questionnaire** -Acceptability of routine antenatal contraceptive counselling and provision as well as LARC uptake and barriers and facilitators to antenatal counselling	-Delivering antenatal contraception at 22 weeks by midwives is feasible and acceptable.
Chen (2022)				NICU in California, USA	Postpartum with NICU infant	16	**Qualitative, interview** -Assessed patient preferences and experiences of peripartum contraceptive counseling	-Patient experiences varied from brief to persistent, informative, and pushy. Trust was important. Felt like a task rather than something participants wanted to discuss.
Congdon (2020)				Pediatric primary care clinic, USA	Postpartum with preterm delivery	41	**Qualitative, interview** -Assessed preference for timing and setting for contraceptive counseling within pediatric setting	-Individuals who delivered prematurely identified additional barriers stemming from their unique postpartum circum- stances. They had fewer third trimester visits, when they expected postpartum planning might have occurred. Reported not wanting to leave hospital for their own postpartum visit and concerned with infants and not themselves.
Fagan (2009)				Community Family Medicine clinic at well-child check in Asheville, NC, USA	No	100	**Cross-sectional, survey** -Assessed comfort and acceptability of discussing contraception with infant provider	-Most would be “very comfortable’ or “somewhat comfortable” discussing contraception with baby’s pediatrician.
Glasier (1996)				Edinburgh, Scotland	No	174	**Cross-sectional, qualitative** -Assessed satisfaction with timing, content of contraceptive counseling, and level of understanding of discussion as well as health outcomes	-Unable to demonstrate in this study a statistically significant relationship between short interpregnancy intervals and poor contraceptive advice.
Harris (2020)				Combined pediatric faculty and resident ambulatory clinic, USA	No	346	**Qualitative, survey and interview** -Assessed postpartum contraceptive knowledge, behavior, and acceptability of pediatrician-delivered contraceptive counseling	-Participants reported willingness to engage in contraceptive counseling discussions with their child’s health care provider.
Henderson (2016)				Chicago, IL, USA	No	32	**Qualitative, interview** -Assessed acceptability and preferences for contraceptive counseling in postpartum period, and earlier than 6 weeks	**-** Participants appreciate support care in the postpartum period but require flexibility in timing and location of visit, and prefer access to birth control as early as possible after delivery if a woman desires it, including at delivery or discharge from the hospital. -Varying views about discussion and provision of contraception at the Well-Baby Visit.
James (2018)				Queensland, Australia	Aboriginal Australians	17	**Qualitative, interview** -Assessed preferences for content and timing of contraceptive counseling in the peripartum period	-Preferences were diverse, inducing for timing, setting, and type of contraceptive counseling materials. -Most preferred antenatal exposure of contraceptive counseling with reinforcement during the postpartum period.
Jarvis (1996)				Wigan, England	No	122	**Cross-sectional, survey** -Assessed acceptability of provision and timing of contraceptive counseling in the immediate postpartum period	-Most prefer discussing contraception before hospital discharge but not immediately after birth.
Leaverton (2015)				Providence, RI, USA	Mothers of infants in NICU	95	**Cross-sectional, sruvey** -Assessed contraceptive plans and preferences for obtaining contraceptive counseling in family planning clinic near NICU	-Most would attend family planning clinic, but only if was in NICU.
Mann (2019)				South Carolina, USA	No	25	**Qualitative, interview** -Assessed pt experiences with immediate pp LARC counseling	-Dissatisfaction with approaches to contraceptive counseling, including over-emphasis of LARC methods; receiving inadequate information about their contraceptive options; timing of immediate post-partum contraceptive counseling (while in labor); barriers to LARC removal.
Pearlman Shapiro (2022)				Bronx, NYC, USA	No	20	**Qualitative, interview** -Preferences for timing and content of contraceptive counseling in relationship to breastfeeding plans	-Most decision-making regarding contraception relied on the personal experiences of the participants and their friends and family. -Dissatisfaction with coercion toward LARC postpartum. -Reinforce that the medical establishment needs to find a way to reframe the conversation around birth spacing to focus on the benefits to the newborn and maternal health.
Roque (2022)				Academic Medical Center in Cleveland OH, USA	Inpatient Adolescents	12	**Qualitative, interview** -Patient preferences for timing of contraceptive counseling in the peripartum period	-Only a minority of teens felt their health care providers played a key role in counseling and decision making about contraception and instead were influenced by social networks more. -Study participants also reported being disappointed in health care providers for missed opportunities to discuss contraceptive options both prior to and during their index pregnancy.
Sober (2017)				Philadelphia, PA, USA	Adolescent postpartum patients	30	**Qualitative, interview** -Preferences for peripartum contraceptive counseling	-Optimally, contraceptive counselling would be provided by a physician (60%) and begin antepartum (80%). -Many amenable to the idea of multiple modalities (including provision of written or video information as well as referral to online resources) as long as in-person contraceptive counselling was the mainstay. -Reasons for feeling comfortable with the provider were that they allowed the subject to make her own decision and did not pressure her to choose a specific method or that they did not find the topic embarrassing.
Sundstrom (2018)				Southeastern USA postpartum outpatient clinic	No	47	**Qualitative, focus group** -Preferences for content and timing of contraceptive counseling in postpartum period	-Participants indicated that they trusted their healthcare provider’s advice but prioritized personal experience and autonomy in decisions about contraception -Participants were interested in receiving resources and advice about a variety of methods. -Participants expressed a preference for relationship-centered care, in which healthcare providers listened, took time to individualize their approach to care through rapport-building, and engaged women in shared decision-making about contraceptive use through open communication, reciprocity, and mutual influence. -According to participants, mutual trust and respect started with listening. Participants who could ask their healthcare providers questions via email or text message were the most satisfied with their care. -Many participants desired the opportunity to build a strong relationship with a healthcare provider. Relationship-centered care relied on healthcare providers treating each patient as an individual.
Sznadjer (2019)				Baltimore, MD, USA	No	17	**Qualitative, interview** -Preferences and experiences of contraceptive counseling in peripartum period	-Participants wanted comprehensive, objective information early and often during antepartum contraceptive counseling; autonomy in their contraceptive decision making to make internally motivated decisions; and those who reported feeling pushed during counseling were critical of their experiences.
Taylor (2022)				Sydney, AU	Pregnant people with live birth less than 18 months prior to conception of current pregnancy	20	**Qualitative, interview** -Experiences with contraceptive counseling in the peripartum period	-Some felt the antenatal period was most appropriate as they would be too overwhelmed in the immediate postpartum period to take on any further information. In contrast, others felt that postpartum was the ideal time to discuss these topics, as it was when they would be most receptive to the counselling.
Thiel de Bocanegra (2020)				California, USA	Patients with recent preterm birth	35	**Qualitative, interview** -Experiences with timing and frequency of contraceptive counseling, quality of patient-provider interaction, context in which contraceptive counseling was framed, contraceptive use and experiences, system barriers to contraception use	-Participants reported providers’ delivery of contraceptive counseling was influential to their quality of care. -Participants reported feeling uncomfortable discussing their concerns or following up with questions with providers who seemed rushed or impatient during the visit. –Those who felt pressured to decide on a contraceptive method in the hospital through repeated or insistent questioning either did not initiate or discontinued usage of their contraceptive method. -Participants felt more involved in the decision making if these conversations about contraceptive choices occurred over several prenatal visits or group prenatal care sessions.
Walker (2021)				UK	Patients receiving midwifery care	227 survey, 10 interviews	**Qualitative, survey** -Preference for contraceptive care in the peripartum period	-A majority reported interest in receiving contraceptive advice from midwives, during pregnancy (56%) and postnatally (63%), although approximately 30% of women indicated that they did not wish to receive advice.
Williams (2019)				Iowa City, IA, USA	No	304	**Cohort, survey** -Patient preference for timing of contraceptive counseling as well as readiness and knowledge about contraception before hospital discharge	-64% of prenatal and 63% of postpartum respondents felt that postpartum contraception should be discussed during the second or third trimester compared with 14% and 13, respectively, indicating it should be discussed at the 6-week postpartum visit.
Wong (2022)				Illinois, USA	Patients receiving care in a Catholic setting	21	**Qualitative, interview** -Patient experiences with postpartum contraception in catholic settings	-Patients and providers agreed that lack of hospital transparency meant patients were unable to make fully informed decisions regarding their family planning methods during the vulnerable postpartum period. In the current study, patients expressed frustration when they were turned away from receiving care.
Yee (2011a and b)				Chicago, IL, USA	Minority population	30	**Qualitative, interview** -Assessed contraceptive counseling preferences and experiences in peripartum period	-Negative counseling experiences included feeling ignored or receiving impersonal counseling. -Women described the undertones of coercion they sometimes perceived in contraceptive counseling, particularly when their method choice differed from the provider’s recommendation. -A minority of women described feelings of racial discrimination taking place in their contraceptive counseling experiences.
Yee (2015)				Chicago, IL, USA	Low-income population	57	**Cross-sectional, survey and interview** -Assessed preferences for peripartum contraceptive counseling	-Features of a positive patient-centered counseling experience included learning about multiple methods, being allowed to make an independent choice, feeling that care was individualized, receiving information about risks and side effects, receiving supplementary written information, having health care providers who took time to fully answer questions, and having frequent provider-initiated conversations. -Participants preferred a multimodal approach to contraceptive counseling. -Most recommended frequent, short episodes of contraceptive counseling throughout pregnancy. -Participants recommended reviewing contraceptive options, reassuring them in their decisions, and reinforcing instructions in the immediate postpartum period and again at the postpartum clinic visit.

## Evidence Synthesis

**Fig. 2:**
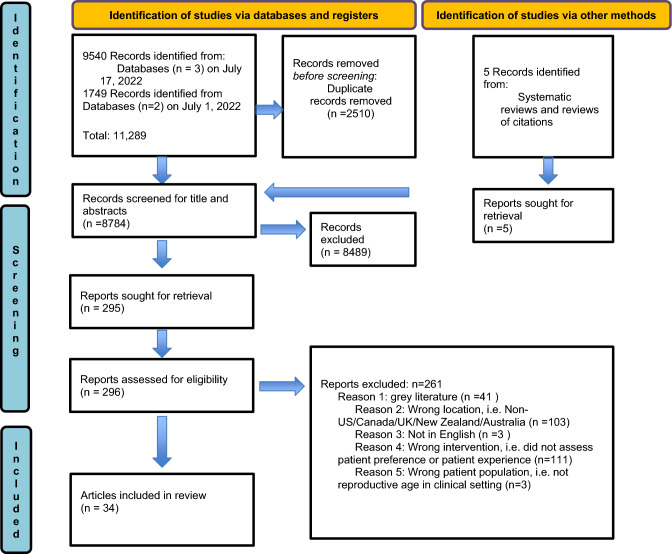
PRIMSA flow chart of included studies in the review Adopted From: Page MJ, McKenzie JE, Bossuyt PM, Boutron I, Hoffmann TC, Mulrow CD, et al. The PRISMA 2020 statement: an updated guideline for reporting systematic reviews. BMJ 2021;372:n71. https://doi.org/10.1136/bmj.n71. For more information, visit: http://www.prisma-statement.org/

**Fig. 3:**
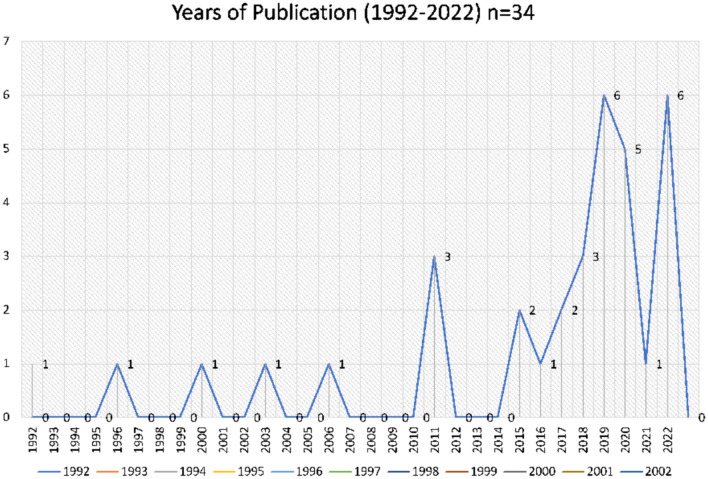
Trend of number of articles published about patient preferences or experiences of contraceptive counseling in the peripartum period (1992–2022)

The literature search yielded 9,540 articles in the first search and an additional 1,704 in the second, for a total of 11,289 articles. Two hundred and ninety-five studies were reviewed in the final full text review and 34 full-text articles (33 studies) were included (Tables 1 and 2), as indicated in the PRISMA chart (Fig. 2). Of the 33 studies included for full extraction, most were based in the United States (n = 24) and published after 2010 (n = 28). Figure 3 shows the trend in publications with the first article to meet criteria published in 1996 and the greatest number of publications in 2019 (n = 6) and 2022 (n = 6).

Of the 33 studies included, 10 included implementation and evaluation of a contraceptive counseling method or protocol, as shown in Table 1. Mapping our key questions onto these studies revealed that they primarily focused on perinatal patients’ experiences of counseling (n = 7), with a small number examining patient preferences (n = 3) and health outcomes (n = 2). Of note, some studies claimed to reveal preferences by reflecting on patient experiences and choices of contraceptive method rather than directly assessing preferences for counseling beforehand. Additionally, studies often assumed that continued use of an effective contraceptive method indicated a positive experience with counseling. In both instances, we concluded that the studies inferred information about counseling experiences and preferences without directly measuring those items, and thus we did not include them in our review.

The remaining 23 studies evaluated existing (often standard of care) contraceptive counseling methods and are listed in Table 2. Many evaluated existing counseling methodologies using qualitative methods to better understand only patient preferences (n = 12 studies) or only patient experiences (n = 4). One study evaluated patient experiences and health outcomes. The remainder (n = 6 studies) utilized surveys and/or interviews to evaluate both patient preferences and experiences with counseling in the perinatal period.

### Key question 1: What are Patients’ Preferences for the Structure and Process of Perinatal Contraceptive Counseling?

Most (n = 22) studies assessed patients’ preferences for the structure, process, and timing of contraceptive counseling in the perinatal period (Tables 1 and 2). Of those that asked patients about timing of counseling (n = 12), most patients wanted counseling in the antenatal period. One group preferred counseling in the second or third trimester, as Sznajder et al. (2019) reported, “early and often.” Sober et al. (2017) assessed pregnant teenagers’ preferences for contraceptive counseling through qualitative surveys and concluded that their participants overwhelmingly (90%) preferred in-person counseling during the antenatal period. Similarly, Gallagher et al. (2019) reported that an intervention of antenatal contraceptive counseling among adolescents in Scotland was preferred over a standard postpartum contraceptive counseling at the 6-week postpartum visit. The 118 adolescents found the timing of the contraceptive discussion with a midwife (at 22 weeks) “about right” (81%) and “very” or “quite helpful” (81%). Those who received standard postpartum counseling also reported a preference for contraceptive counseling in the antenatal period. All studies concluded that counseling should be individualized and flexible during the antenatal period.

Some studies focused on evaluating patient preferences for contraceptive counseling in the postpartum period, specifically at the time of the well-child visit by the infant’s physician (in the first 6 weeks postpartum) or in the hospital after delivery. Studies on pediatrician-provided counseling found that patients were comfortable in this setting: Harris et al. (2020) reported 65% of their respondents were supportive of the idea of receiving counseling at the well-child visit, Fagan et al. (2009) reported that 87% of their participants expressed comfort talking to a pediatrician about contraception, and Kumaraswami et al. (2018) reported that 95% of participants were comfortable talking about contraception at a well-child visit. Henderson et al. (2016) however, reported mixed results: while the majority of participants were in favor of receiving contraceptive services at the well-baby visit, some felt that these visits should be wholly focused on the baby. The authors concluded that approaches emphasizing flexibility and convenience would allow the greatest number of patients to utilize postpartum contraceptive services.

Many studies reported that patients expressed opposition to discussing contraception during labor or addressing the topic for the first time in the hospital. Studies that assessed counseling in the inpatient setting suggested that most patients preferred counseling outside the hospital setting. For example, Mann et al. (2019) found that some individuals objected to receiving contraceptive counseling in the hospital because they were in labor and/or already had a plan for postpartum contraception.

Only one study evaluated preferences for content and/or delivery method of contraceptive counseling: Staley et al. (2002) assessed acceptability of a LARC-first video in a RCT. The researchers found that 95.2% of patients in the intervention arm regarded video-based counseling as acceptable. However, they did not query participants in the control group about preferences for contraceptive counseling content or delivery.

Finally, some studies that addressed KQ1 focused on the preferences of specific populations. Four studies assessed preferences for contraceptive counseling via qualitative interviews with postpartum patients who had experienced preterm deliveries. These studies found that perinatal patients with preterm infants were typically focused on their infants’ health and had stressors particular to their situation. (Chen et al., 2022; Leaverton et al., (2016); Congdon et al., (2020); Thiel de Bocanegra et al. (2020)). Patients were willing to receive information about contraception, but they tended to be focused on the needs of their infant. Patients did appreciate pediatric expertise about the intersection of breastfeeding, contraception, and preterm infants’ growth.

Thiel de Bocanegra et al. (2020) stands out among the studies that evaluated KQ1, as it provided sociodemographic details for both individual and systems-level factors that contextualized patients’ preferences. The researchers identified that patients’ preferences for contraceptive counseling were associated with their age, birthing experiences, and childbearing goals. Most patients (n = 23 out of 35) preferred for conversations around postpartum contraception to occur over several antenatal visits, with time in between visits to consult family and friends about their experiences with contraceptive methods.

Finally, some studies addressing KQ1 focused on specific demographic groups. James et al. (2018) assessed preferences of aboriginal Australians and found a diversity of preferences, including suggestions about printed material, timing, and group versus one-on-one counseling. Sober et al. (2017) assessed timing of postpartum contraceptive counseling for adolescents and found that teens preferred counseling delivered by a physician in the antenatal period. Yee and Simon (2011a) found that low-income, minority perinatal patients preferred frequent, short episodes of counseling that included multimodal approaches. Importantly, these studies showed that patients wanted counseling for which they had a “feeling that one’s health care provider was caring, empathetic, truthful, and interested in them.” (Yee & Simon, 2011b).

### Key Question 2: How is the Delivery of Contraceptive Counseling in the Perinatal Period Associated with Patient Experience of Counseling?

Of the 19 studies that addressed KQ2, eight implemented or evaluated a new contraceptive counseling approach (Table 1) and 11 evaluated patient experiences of existing counseling (Table 2). Two main themes emerged with respect to KQ2. First, greater satisfaction and quality were reported when providers were flexible and provided individualized counseling. For example, Sundstrom et al. (2018) and Sznajder et al. (2020) found that patients experienced higher satisfaction when counseling “supported a woman’s individual needs and desires.” (Sznajder et al., 2020). Second, individuals were most critical of their counseling experiences when they reported feeling pushed toward particular contraceptive methods, or when they felt providers weren’t interested in their individual needs. For example, Yee and Simon’s (2011b) qualitative study with low-income, minority perinatal patients (n = 30) found that one-third of participants (n = 10) described “feeling coerced” or experiencing “racially-based discrimination in counseling,” with “pushy” providers associated with negative counseling interactions.

Johnson et al. (2003) compared satisfaction and effectiveness of standard counseling against counseling with additional written educational materials and found no difference in satisfaction between the groups. Those that received the written material were more likely to report it contributed to their ultimate contraceptive decision than the control group (p < 0.01). Moniz et al. (2022) evaluated a new toolkit-based implementation of immediate postpartum LARC counseling and provision and found poor patient satisfaction. Proctor et al. (2006) compared patient satisfaction of physician–patient counseling against either written literature or educational videos after randomizing patients into three groups to receive either physician counseling, written materials, or an educational video. While patient satisfaction was high across all three groups (> 90% satisfaction), the authors noted a statistically significant trend towards increased satisfaction with provider-delivered counseling (p < 0.05) compared to the other arms. Additionally, across all arms, African American (98.2%) and Hispanic (93.5%) patients were more satisfied than “Caucasian” (83.3%) patients (p = 0.026) and satisfaction with contraceptive counseling decreased with patient age.

Of the eight studies that implemented a new counseling approach while assessing KQ2, three assessed patient experience of counseling in the immediate postpartum period, two at a postpartum well baby visit, and three at antenatal visits. In the antenatal period, Haider et al. (2020) and Kumaraswami et al. (2018) both assessed patient experience of contraception at a well-child visit. Haider et al. attempted to evaluate if co-locating contraceptive services at a well-baby visit influenced patient experience of counseling. Although uptake of the visits was low, those who accepted the visit reported a high rate of satisfaction (80%), and 64% said they would recommend a linked contraceptive visit with a well-baby care visit to a friend. It was noted that scheduling the co-visits ahead of time, rather than at the well-baby visit, would make it easier. The individuals who did not accept the visit reported not wanting to see a new provider, not wanting their children present at a contraceptive appointment, not wanting to extend the length of the visit, being tired, and not wanting to stay at the clinic.

Three studies assessed satisfaction of counseling and contraceptive use in the antenatal period. Reyes-Lacalle et al. (2020) randomized patients at the 30-week prenatal visit to standard (24–48 h after delivery and 6 week postpartum) versus standard counseling with supplemental “holistic” contraceptive counseling (provided in person at 35 weeks of pregnancy with printed and online written information, supplemented by a short message service (SMS reminder at week 37 of pregnancy and an in-person meeting with a counselor at 2 weeks after delivery.) Researchers found higher satisfaction with the experience of contraceptive counseling in the intervention group. Similarly, Smith et al. (2002) randomly assigned over 600 patients attending antenatal clinics in Edinburgh, Scotland to receive standard advice (provided postpartum in the hospital) or expert contraceptive advice (individualized contraceptive care provided antenatally by a family planning specialist nurse). Sixty-seven percent of participants in the intervention group who responded to a 16-week postpartum survey (n = 171) said they found the opportunity to discuss postpartum contraception in the antenatal period “helpful.”

Of the 19 studies that addressed KQ2 (association of contraceptive counseling and patient experience), eight did so without also addressing either KQ1 (patients’ preferences for the structure and process of perinatal contraceptive counseling) or KQ3 (association between patient experiences of perinatal contraceptive counseling and patient health outcomes); nine assessed both KQ1 and KQ2; and two assessed KQ2 and KQ3 in the same study. Of the 11 articles that assessed the impact of routine contraceptive counseling (Table 2), seven assessed KQ1 and KQ2, one assessed KQ2 and KQ3, and three assessed only KQ2. The one study that correlated patient experience of counseling with health outcomes (which will be discussed under KQ3), found that over 50% of the patients interviewed reported negatively about some aspect of the counseling they received, most commonly the limited discussion of methods besides oral contraceptive pills and condoms (Glasier et al., 2017). Of the seven studies that assessed patient preferences for counseling in conjunction with reporting on patient experiences of counseling, all reported varied patient experiences of counseling, with the most positive experiences reported by patients who received counseling about a variety of methods and from providers whom they came to trust.

### Key Question 3: What are the Associations between Patient Experiences of Perinatal Contraceptive Counseling and Patient Health Outcomes?

Only three studies investigated the associations between patient experiences of contraceptive counseling and health outcomes; two were RCTs (Frarey et al., 2019; Smith et al., 2002) and one was a retrospective cross-sectional study (Glasier et al., 1996). None evaluated KQ1 and KQ3 in the same study; thus, none correlated whether receiving counseling concordant with patient preferences led to a lowered risk for adverse health outcomes. Rapid repeat pregnancy, defined as repeat pregnancy within one year of delivery, was the primary health outcome measured in all three studies. None of these studies assessed whether rapid repeat pregnancy was directly associated with negative infant or maternal health outcomes, such as gestational diabetes, preterm birth, low birth weight, or small-for-gestational age (SGA).

Frarey et al. (2019) compared standardized postpartum counseling for adolescents with additional counseling incorporating information on healthy birth spacing and LARC methods. They found no difference in pregnancy rates or in satisfaction rates at 6 or 12 months between the two arms. Smith et al (2002)., found no association between the receipt of expert contraceptive advice and repeat pregnancy rates at 1 year. The researchers concluded that although peripartum patients in all centers said they found the opportunity to discuss contraception antenatally useful, it had very little effect on subsequent pregnancy rates.

Glasier et al. (1996) conducted a qualitative cross-sectional study to determine what advice peripartum patients received about postpartum contraception and their satisfaction with this counseling, and to assess the relationship between contraceptive advice and short interpregnancy intervals. The patient experience measures they assessed included satisfaction with the timing of contraceptive counseling, satisfaction with the content of counseling, whether the counseling was helpful/unhelpful, and level of understanding of discussion. Up to 84% of the sample discussed contraception with a midwife on the postnatal ward, but discussion was often felt to be brief, limited, and frequently provided as the patient was leaving the hospital. Almost all individuals reported discussing contraception with their general practitioner at the postnatal check, but a significant number felt that the choice of method was limited to condoms or pills. Based on their finding that almost half of study participants reported negative experiences with the contraceptive counseling they received on the postnatal ward, the authors concluded that the postnatal ward is not an appropriate setting for discussion about future contraception. The researchers did not find a statistically significant relationship between contraceptive counseling with “poor” compared to “helpful” satisfaction ratings and short interpregnancy intervals.

### Contextual Considerations

The included studies assessed diverse sets of patients, providers, and patient-provider dyads. Fourteen of the articles focused on specific populations. Importantly, however, few of the articles discussed the impact of race and ethnicity on contraceptive counseling preferences and experiences, despite established differences among racial/ethnic groups in the selection of contraceptive methods (Shih et al., 2011) and preferences for contraceptive characteristics. (Callegari et al., 2016; Jackson et al., 2015).

Moreover, no study explicitly included transgender or trans-expansive patients, and none of the studies noted that they focused on cis-gender females. Just as all studies omitted discussion about non-cis-gender individuals, 16 studies acknowledged the race/ethnicity of patients but did not report on differences in preferences and experiences of contraceptive counseling by race/ethnicity in the results.

Yee and Simon (2011b) explicitly focused on perceptions of coercion, discrimination, and negative experiences in postpartum contraceptive counseling among racial/ethnic minority patients and found that, “receiving impersonal, hurried, incomplete, or uncaring counseling turned some [minority patients] away from using recommended effective contraception methods.” Similarly, Congden et al. (2020) found that several study participants who were Black, indigenous and people of color (BIPOC) and/or low-income reported feeling judged by providers and coerced into choosing more effective contraceptive methods. One of the main findings in Pearlman Shapiro et al.’s (2022) study was that “especially socioeconomically disadvantaged women of color remain distrustful of medical professionals when it comes to contraception. This is especially apparent when it comes to methods that are not under a woman’s control and require implantation by a medical provider.” Conversely, Proctor et al. (2006) found that satisfaction with contraceptive counseling was highest among people of color in their sample. They attributed this to “Caucasian women” having higher expectations from the health care system. However, they did not further explore this finding, which could also be due to the general experience of inequity by people of color or the lack of specificity of their question to assess reasons for dissatisfaction with counseling.

## Discussion

Across the studies that examined patients’ preferences about contraceptive counseling in the perinatal period, common themes included: (1) preference for receiving contraceptive counseling at some point during the antenatal period before the inpatient hospital experience (KQ1); (2) preferences for direct provider-patient discussion as opposed to video links and written material (KQ1); (3) rapport with providers and discussion of multiple contraceptive options as an important component of quality counseling (KQ1); (4) acceptability of discussing contraception after delivery in the hospital setting, but varied experiences with discussions in this setting (KQ1 and KQ2); (5) negative counseling experiences in the context of perceived pressure to use a method and, for BIPOC patients, experience of bias and discrimination (KQ2); (6) openness to contraceptive counseling with a pediatrician during the postpartum period, but difficulty balancing attention to infant’s need with their own during this time (KQ1 and KQ2); and (7) no clear association between patient experience of counseling and rapid repeat pregnancy rate (KQ3). Overall, our review found a consistent preference for counseling that is tailored to individuals needs and circumstances, and that is non-directive.

The themes identified for KQ1 and KQ2 are consistent with approaching contraceptive counseling from a patient centered, reproductive justice framework, as outlined in the ACOG guidelines around contraceptive counseling (ACOG, 2022), and are also consistent with the prior review by Fox et al. addressing preferences for contraceptive counseling generally. Our findings build on this analysis as Fox et. al., only reviewed studies up until 2016; as shown in Fig. 3, the number of studies related to patient preferences for contraceptive counseling has expanded significantly since that time. Additionally, our analysis provides more detail about preferences specifically in the perinatal period, which shows a clear relationship between explicit interventions and initiatives integrating patient centered care and eliminating bias and paternalism in reproductive health care. This data suggests that patients prefer flexibility and individualized, tailored counseling and perceive coercion and bias as negative experiences. Further, the findings specifically about the timing of counseling and the acceptability of contraceptive counseling in the context of well child care are both specific to the perinatal period.

As mentioned previously, a common cause for exclusion from our search was when studies assessed the relationship between contraceptive counseling and patient choice of contraceptive method without addressing any aspect of patient preferences for or experiences of counseling. We excluded 111 out of 295 articles for this reason. This high number of excluded articles reflects the problematic assumptions that choice of an effective method is the ultimate positive outcome of contraceptive counseling in the perinatal period. While increased patient satisfaction may, in some circumstances, contribute to the selection of effective contraception, the use of method satisfaction as a proxy for satisfaction with counseling reflects a bias about the goal of counseling, suggesting that the goal should be getting people to choose certain methods instead of meeting people’s informational and decision support needs. In fact, this speaks to the bias of paternalism in contraceptive counseling in clinical care and research, which has been addressed more and more frequently as the literature has evolved.

The lack of studies answering KQ3 and the negative fundings in the studies identified do not provide a clear answer to how quality contraceptive counseling may influence SIP or other health outcomes.. Importantly, the fact that no study addressed both KQ1 and KQ3 highlights the disconnect between research on the health impacts on counseling and research on experience of care. Research in the context of non-pregnant patients has shown that patient-centered counseling is associated with contraceptive continuation and use of contraception. This indicates that future work to leverage contraceptive care to optimize pregnancy outcomes would benefit from attention to the results of KQ1 and KQ3 in designing interventions that meet perinatal patients’ needs. We also note that studies we reviewed for KQ3 defined short interval pregnancy as delivery within 18 months of previous pregnancy. However, the data from Congdon et al., 2022 suggests that only short interval pregnancies within 6 months confer negative health outcomes. Future counseling interventions in the perinatal period should provide patients with accurate, understandable information about the potential risks of SIP using this best evidence so that they can be supported to make informed decisions that reflect their preferences.

Limitations of our scoping review include that, as mentioned, the value of information from many studies was limited due to assumptions about what outcomes were important and measured. This is further illustrated by the large number of articles excluded from this review. This indicates a need to shift research in the field of contraceptive counseling toward a more patient-centered lens. In addition, because we elected to focus on contraceptive counseling experiences and interventions in the United States, Canada, Europe, the UK, New Zealand, and Australia, our findings cannot necessarily be extended to other settings. Our review also did not include articles published in non-English languages, which similarly limits generalizability. Finally, like any scoping review, our analysis of the literature is limited by the primary literature itself; our findings are only as reliable as the methods used in the primary studies.

In conclusion, our review supports the provision of non-directive perinatal contraceptive counseling focused on patients’ needs and circumstances that is centered around the individual patient’s needs and is not a general one-size fits all model. This approach requires flexibility and willingness to accommodate individuals’ preferences, even if they change over time. While some patients may benefit from, and appreciate the use of, written educational materials such materials should be used to supplement, rather than replace, counseling offered by a trusted healthcare provider as studies found a preference for positive rapport from an in person provider when discussing contraceptive counseling. A willingness to accommodate patients’ preferences will facilitate rapport between patients and providers and is an important component of patient-centered care. Avoiding coercive counseling, and perceptions thereof, is also crucial for providers offering perinatal contraceptive counseling, particularly those serving populations that have experienced reproductive violence and neglect from institutional healthcare. Findings from this scoping review can be used to develop patient-centered counseling interventions and validated evaluation tools that center patient experience and preferences as primary outcomes. Developing innovative approaches to support quality contraceptive counseling and provision can optimize health outcomes and support the reproductive autonomy of pregnant and recently pregnant individuals, which can also increase trust in reproductive health providers (Dehlendorf et al., 2013) and access to reproductive health care (Gomez & Wapman, 2017). If patient-centered care is the goal, it is critical that ongoing and future research prioritize eliciting patients’ preferences for and experiences with that care alongside associations with health outcomes.

## Data Availability

Not applicable.

## Appendix 1

### Search Strategy

**Table:**

Database	Search strategy
PubMed (1966-)	(Contraception[Mesh] OR “Contraception Behavior”[Mesh] OR “Family Planning Services”[Mesh] OR contraception[tiab] OR contraceptive[tiab] OR contraceptives[tiab] OR “birth control”[tiab] OR larc[tiab] OR “long acting reversible contraceptive”[tiab] OR “intrauterine device”[tiab] OR “intrauterine devices”[tiab] OR iud[tiab] OR iuds[tiab] OR “nuva ring” or “nuvaring” or “vaginal ring” OR “barrier method”[tiab] OR “barrier methods”[tiab] OR “Contraceptive Devices”[Mesh] OR “Contraceptive Agents, Female”[Mesh] OR levonorgestrel[tiab] OR “plan b”[tiab] OR “morning after pill”[tiab]) AND (“Postpartum Period”[Mesh] OR “Peripartum Period”[Mesh] OR “Postnatal Care”[Mesh] OR “Prenatal Care”[Mesh] OR peripartum[tiab] OR postpartum[tiab] OR antepartum[tiab] OR antenatal[tiab] OR prenatal[tiab] OR antenatally[tiab] OR prenatally[tiab] OR postnatal[tiab]) AND (perception[tiab] OR perceptions[tiab] OR perceived[tiab] OR perceiving[tiab] OR perceive[tiab] OR perceives[tiab] OR experiences[tiab] OR preferences[tiab] OR preference[tiab] OR preferred[tiab] OR perspectives[tiab] OR perspective[tiab] OR beliefs[tiab] OR feeling[tiab] OR feelings[tiab] OR attitudes[tiab] OR attitude[tiab] OR satisfaction[tiab] OR satisfied[tiab] OR comfortable[tiab] OR prioritize[tiab] OR prioritized[tiab] OR trust[tiab] OR trusting[tiab] OR mistrust[tiab] OR distrust[tiab] OR experience[tiab] OR “return rate”[tiab] OR “return rates”[tiab] OR control[tiab] OR discrimination[tiab] OR “patient-doctor communication”[tiab] OR “doctor-patient communication”[tiab] OR continuity[tiab] OR choice[tiab] OR choices[tiab] OR preterm[tiab] OR preeclampsia[tiab] OR “gestational diabetes”[tiab] OR atony[tiab] OR hemorrhage[tiab] OR hemorrhaging[tiab] OR “placental implantation”[tiab] OR “low birth weight”[tiab] OR “infant mortality”[tiab] OR complications[tiab] OR “unintended pregnancy”[tiab] OR “unintended pregnancies”[tiab] OR client-centered[tiab] OR person-centered[tiab] OR “rapid repeat pregnancy”[tiab] OR “Attitude to Health”[Mesh] OR “Physician–Patient Relations”[Mesh])
Embase (1947-)	(‘female contraceptive device’/exp OR ‘contraceptive agent’/exp OR ‘family planning’/exp OR ‘contraception’/exp OR ‘contraceptive behavior’/exp OR contraception:ab,ti OR contraceptive:ab,ti OR contraceptives:ab,ti OR “birth control”:ab,ti OR larc:ab,ti OR “long acting reversible contraceptive”:ab,ti OR “intrauterine device”:ab,ti OR “intrauterine devices”:ab,ti OR iud:ab,ti OR iuds:ab,ti OR “barrier method”:ab,ti OR “barrier methods”:ab,ti OR “vaginal ring”:ab,ti OR “nuva ring”:ab,ti OR nuvaring:ab,ti OR levonorgestrel:ab,ti OR “plan b”:ab,ti OR “morning after pill”:ab,ti) AND (‘perinatal period’/exp OR ‘postnatal care’/exp OR ‘prenatal care’/exp OR peripartum:ab,ti OR postpartum:ab,ti OR antepartum:ab,ti OR antenatal:ab,ti OR prenatal:ab,ti OR antenatally:ab,ti OR prenatally:ab,ti OR postnatal:ab,ti) AND (perception:ab,ti OR perceptions:ab,ti OR perceived:ab,ti OR perceiving:ab,ti OR perceive:ab,ti OR perceives:ab,ti OR experiences:ab,ti OR preferences:ab,ti OR preference:ab,ti OR preferred:ab,ti OR perspectives:ab,ti OR perspective:ab,ti OR beliefs:ab,ti OR feeling:ab,ti OR feelings:ab,ti OR attitudes:ab,ti OR attitude:ab,ti OR satisfaction:ab,ti OR satisfied:ab,ti OR comfortable:ab,ti OR prioritize:ab,ti OR prioritized:ab,ti OR trust:ab,ti OR trusting:ab,ti OR mistrust:ab,ti OR distrust:ab,ti OR experience:ab,ti OR “return rate”:ab,ti OR “return rates”:ab,ti OR control:ab,ti OR discrimination:ab,ti OR “patient-doctor communication”:ab,ti OR “doctor-patient communication”:ab,ti OR continuity:ab,ti OR choice:ab,ti OR choices:ab,ti OR preterm:ab,ti OR preeclampsia:ab,ti OR “gestational diabetes”:ab,ti OR atony:ab,ti OR hemorrhage:ab,ti OR hemorrhaging:ab,ti OR “placental implantation”:ab,ti OR “low birth weight”:ab,ti OR “infant mortality”:ab,ti OR complications:ab,ti OR “unintended pregnancy”:ab,ti OR “unintended pregnancies”:ab,ti OR client-centered:ab,ti OR person-centered:ab,ti OR “rapid repeat pregnancy”:ab,ti OR ‘attitude to health’/exp)
Popline (1970-)	(contraception OR contraceptive OR contraceptives OR “birth control” OR larc OR “long acting reversible contraceptive” OR “intrauterine device” OR “intrauterine devices” OR iud OR iuds OR “barrier method” OR “barrier methods” OR “vaginal ring” OR “nuva ring” OR nuvaring OR levonorgestrel OR “morning after pill”) AND (peripartum OR postpartum OR antepartum OR antenatal OR prenatal OR antenatally OR prenatally OR postnatal) AND (perception OR perceptions OR perceived OR perceiving OR perceive OR perceives OR experiences OR preferences OR preference OR preferred OR perspectives OR perspective OR beliefs OR feeling OR feelings OR attitudes OR attitude OR satisfaction OR satisfied OR comfortable OR prioritize OR prioritized OR trust OR trusting OR mistrust OR distrust OR experience OR “return rate” OR “return rates” OR control OR discrimination OR “patient-doctor communication” OR “doctor-patient communication” OR continuity OR choice OR choices OR preterm OR preeclampsia OR “gestational diabetes” OR atony OR hemorrhage OR hemorrhaging OR “placental implantation” OR “low birth weight” OR “infant mortality” OR complications OR “unintended pregnancy” OR “unintended pregnancies” OR client-centered OR person-centered OR “rapid repeat pregnancy”)

## Appendix 2

### PRISMA-S Checklist Peripartum Review

**Table:**

Section/topic	#	Checklist item	Reported on page #	Reported in abstract	Reported in Suppl
DATABASES					
Databases	1	Describe fully all databases searched	1–2	x	x
Database name	1A	Name each individual database searched	1–2	x	x
Interface	1B	State the platform, interface, provider, vendor, or host system for each database searched	1–2		x
Dates of Coverage	1C	List the dates of coverage for each database searched	1–2		x
Multidatabase Searching	1D	If databases were searched simultaneously through a single interface, state the name of the interface and list all of the databases included and their dates of coverage individually	na		x
ADDITIONAL INFORMATION SOURCES					
Additional information sources	2	Describe all other information sources and methods used as part of the search process	1–2		
Online resources	2A	List any trials registries, web search engines, specific web sites, conference proceedings, or other resource searched, including their dates of coverage	n/a		
Manual searching	2B	If manual searching or handsearching was conducted, list the names of all handsearched sources, including the dates of coverage	2		
Citation searching	2C	Indicate whether cited references or citing references were examined, and describe any methods used for locating cited/citing references (e.g., manual search; name, platform, and dates of coverage for any citation index used; email alerts)	2		
Text analysis methods	2D	Describe or cite pre-defined individual or sets of records and/or software or applications used for textual analysis to derive search terms or for other automated text-mining techniques	n/a		
Contacts	2E	Indicate whether additional studies or data were sought by contacting authors, experts, manufacturers, or other contacts	n/a		
Other methods	2F	Describe any additional supplementary search methods used	n/a		
LIMITS AND RESTRICTIONS					
Limits and restrictions	3	Specify that no limits were used or describe any limits or restrictions applied to each search and provide justification for their use, including: a. Date or time period; b. Language; c. Publication status; d. Human or Organism; e. Study design; f. Database subsets; g. Pre-specified cut-off points for inclusion of search results (e.g. from internet searches); h. Other restriction	2–3		x
FILTERS AND PRIOR WORK					
Search filters	4	Indicate and cite when published search filters or hedges were used for any search, and whether they were modified or adapted from their published versions	n/a		
Prior work	5	Indicate and cite when search strategies from other literature reviews were adapted or reused for part or all of the search	n/a		
FULL SEARCH STRATEGIES					
Full search strategies	6	Include the search strategies for each database and resource, copied and pasted exactly as run, including any updates	Appendix 1		x
DATES OF SEARCHES					
Dates of searches	7	For each source, provide the date when the search and any subsequent update(s) occurred		x	x
UPDATES					
Updates	8	Report the methods used to update the search(es)	n/a		
SEARCH DESIGNER(S)					
Search designer(s)	9	Describe who designed and/or executed the search	2–3		
PEER REVIEW					
Peer review	10	Describe any search peer review process	n/a		
MANAGING RECORDS					
Total records	11	Document the total number of references identified from each database and additional information source	Figure 1		x
Deduplication	12	Describe the processes and any software used to deduplicate records from multiple database or other resource searches	n/a		x
Records screened	13	Document the number of records for screening after duplicates removed	Figure 1		x

Preferred Reporting Items for Systematic review and Meta-Analysis Searches (PRISMA-S) 2019 statement Rethlefsen ML, Koffel JB, Kirtley S, Waffenschmidt S, Ayala AP, PRISMA-S Group

Version 1.0, released March 20, 2019

## Appendix 3

### Preferred Reporting Items for Systematic Reviews and Meta-Analyses Extension for Scoping Reviews (PRISMA-ScR) Checklist

**Table:**

SECTION	ITEM	PRISMA-ScR CHECKLIST ITEM	REPORTED ON PAGE #
TITLE			
Title	1	Identify the report as a scoping review	Title page
ABSTRACT			
Structured summary	2	Provide a structured summary that includes (as applicable): background, objectives, eligibility criteria, sources of evidence, charting methods, results, and conclusions that relate to the review questions and objectives	1
INTRODUCTION			
Rationale	3	Describe the rationale for the review in the context of what is already known. Explain why the review questions/objectives lend themselves to a scoping review approach	1
Objectives	4	Provide an explicit statement of the questions and objectives being addressed with reference to their key elements (e.g., population or participants, concepts, and context) or other relevant key elements used to conceptualize the review questions and/or objectives	1
METHODS			
Protocol and registration	5	Indicate whether a review protocol exists; state if and where it can be accessed (e.g., a Web address); and if available, provide registration information, including the registration number	1, line 58
Eligibility criteria	6	Specify characteristics of the sources of evidence used as eligibility criteria (e.g., years considered, language, and publication status), and provide a rationale	2
Information sources*	7	Describe all information sources in the search (e.g., databases with dates of coverage and contact with authors to identify additional sources), as well as the date the most recent search was executed	2
Search	8	Present the full electronic search strategy for at least 1 database, including any limits used, such that it could be repeated	Appendix 1
Selection of sources of evidence†	9	State the process for selecting sources of evidence (i.e., screening and eligibility) included in the scoping review	2
Data charting process‡	10	Describe the methods of charting data from the included sources of evidence (e.g., calibrated forms or forms that have been tested by the team before their use, and whether data charting was done independently or in duplicate) and any processes for obtaining and confirming data from investigators	2–3
Data items	11	List and define all variables for which data were sought and any assumptions and simplifications made	3
Critical appraisal of individual sources of evidence§	12	If done, provide a rationale for conducting a critical appraisal of included sources of evidence; describe the methods used and how this information was used in any data synthesis (if appropriate)	n/a
Synthesis of results	13	Describe the methods of handling and summarizing the data that were charted	3–10
RESULTS			
Selection of sources of evidence	14	Give numbers of sources of evidence screened, assessed for eligibility, and included in the review, with reasons for exclusions at each stage, ideally using a flow diagram	Figure 2
Characteristics of sources of evidence	15	For each source of evidence, present characteristics for which data were charted and provide the citations	3
Critical appraisal within sources of evidence	16	If done, present data on critical appraisal of included sources of evidence (see item 12)	n/a
Results of individual sources of evidence	17	For each included source of evidence, present the relevant data that were charted that relate to the review questions and objectives	3–10
Synthesis of results	18	Summarize and/or present the charting results as they relate to the review questions and objectives	3–10
DISCUSSION			
Summary of evidence	19	Summarize the main results (including an overview of concepts, themes, and types of evidence available), link to the review questions and objectives, and consider the relevance to key groups	11
Limitations	20	Discuss the limitations of the scoping review process	12
Conclusions	21	Provide a general interpretation of the results with respect to the review questions and objectives, as well as potential implications and/or next steps	11–13
FUNDING			
Funding	22	Describe sources of funding for the included sources of evidence, as well as sources of funding for the scoping review. Describe the role of the funders of the scoping review	Title page

* Where *sources of evidence* (see second footnote) are compiled from, such as bibliographic databases, social media platforms, and Web sites

† A more inclusive/heterogeneous term used to account for the different types of evidence or data sources (e.g., quantitative and/or qualitative research, expert opinion, and policy documents) that may be eligible in a scoping review as opposed to only studies. This is not to be confused with *information sources* (see first footnote)

‡ The frameworks by Arksey and O’Malley (6) and Levac and colleagues (7) and the JBI guidance (4, 5) refer to the process of data extraction in a scoping review as data charting

§ The process of systematically examining research evidence to assess its validity, results, and relevance before using it to inform a decision. This term is used for items 12 and 19 instead of “risk of bias” (which is more applicable to systematic reviews of interventions) to include and acknowledge the various sources of evidence that may be used in a scoping review (e.g., quantitative and/or qualitative research, expert opinion, and policy document)

*JBI* Joanna Briggs Institute; *PRISMA-ScR* Preferred Reporting Items for Systematic reviews and Meta-Analyses extension for Scoping Reviews

*From:* Tricco AC, Lillie E, Zarin W, O’Brien KK, Colquhoun H, Levac D, et al. PRISMA Extension for Scoping Reviews (PRISMA-ScR): Checklist and Explanation. Ann Intern Med.;169:467–473. 10.7326/M18-0850
